# Portal Venous Drainage Modulates Inflammatory and Apoptotic Responses in a Swine Model of Living Donor Intestinal Transplantation

**DOI:** 10.1111/petr.70210

**Published:** 2025-10-23

**Authors:** Guilherme F. Paganoti, Uenis Tannuri, Alessandro R. Belon, Josiane O. Gonçalves, Suellen Serafini, Raimundo R. N. Guimarães, Felipe Y. Matsushita, Ana Cristina A. Tannuri

**Affiliations:** ^1^ Institute for Children and Adolescents at the University of São Paulo São Paulo Brazil; ^2^ University of São Paulo São Paulo Brazil

**Keywords:** caspase‐3, IL‐1α, immune modulation, ischemia–reperfusion injury, pediatric intestinal transplantation, portal venous drainage

## Abstract

**Background:**

Intestinal failure in children, when unresponsive to rehabilitation, requires intestinal transplantation as the only definitive therapy. In regions with limited availability of deceased donors, living‐donor intestinal transplantation (LDIT) represents an important alternative. The early immunometabolic consequences of venous drainage configuration, however, remain insufficiently defined. Because ischemia–reperfusion injury is central to graft dysfunction, understanding how portal versus systemic venous outflow shapes the immediate postoperative response is essential to guide pediatric strategies.

**Methods:**

A juvenile swine model (*n* = 14) was used to compare portal (*n* = 7) and systemic (caval, *n* = 7) venous drainage after LDIT. Animals were followed for 4 days with serial biochemical, histological, immunohistochemical, and molecular assessments. Analyses included linear mixed‐effects models (LMM) for repeated measures and principal component analysis (PCA) to integrate multivariable data and identify global immunometabolic patterns.

**Results:**

Hepatic and renal function were preserved in both groups. Histology revealed only mild ischemia–reperfusion injury (Chiu/Park grades 1–2), with a trend toward greater lymphocytic infiltration in the systemic group. Caspase immunohistochemistry demonstrated early apoptotic activation in the portal group, which declined by day 4, suggesting a controlled adaptive response. IL‐1α expression was selectively upregulated in intestinal tissue from the portal group, consistent with early mucosal immune activation. PCA confirmed a distinct immunometabolic profile under portal drainage, characterized by balanced inflammation, controlled apoptosis, and trends toward enhanced protein synthesis.

**Conclusion:**

Venous drainage configuration modulates early biological responses after LDIT. Portal drainage was associated with a more regulated immunometabolic profile, supporting the hypothesis that physiological venous outflow promotes mucosal protection and immune balance.

## Graphical abstract

Portal venous drainage in a swine model of living‐donor intestinal transplantation promoted a more balanced immunometabolic profile, with early apoptotic modulation and increased IL‐1α expression, suggesting improved mucosal adaptation compared with systemic drainage.

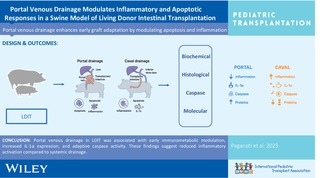

**Abbreviations**
ALP: alkaline phosphatase
ALT: alanine aminotransferase
AST: aspartate aminotransferase
BAX: bcell lymphoma‐2‐associated × protein
Bcl‐XL: b‐cell lymphoma extra large
CXCL10: C‐X‐C motif chemokine ligand 10
GGT: gamma glutamyl transferase
HE: hematoxylin–eosin
HTK: histidine tryptophan ketoglutarate
IL‐17: interleukin 17
IL1‐a: interleukin‐1 alpha
IL1‐b: interleukin‐1 beta
IL‐6: interleukin 6
INF‐gamma: interferon gamma
INOS: inducible nitric oxide synthase
IQR: interquartile range
IRI: ischemia–reperfusion injury
LDIT: living‐donor intestinal transplantation
LMN: linear mixed‐effects model
PC1: principal component 1
PC2: principal component 2
PC3: principal component 3
PCA: principal component analysis
POD: postoperative days
RNA: ribonucleic acid
SD: standard deviation
TNF‐Alpha: tumor necrosis factor—alpha
TPN: total parenteral nutrition

## 1 Introduction

Intestinal transplantation represents a definitive therapeutic option for pediatric patients with intestinal failure refractory to medical and surgical rehabilitation, being the only alternative to lifelong dependence on total parenteral nutrition (TPN) [1, 2, 3, 4, 5]. The procedure can be performed as an isolated intestinal graft or in combination with other organs (multivisceral transplantation), typically utilizing deceased donors. Living‐donor intestinal transplantation (LDIT) accounts for fewer than 3% of all intestinal transplants, primarily due to the absence of standardized surgical protocols and concerns regarding donor safety [6]. Nonetheless, LDIT may offer a feasible alternative in regions where access to deceased donors is limited.

Intestinal transplantation significantly improves survival and quality of life in patients with irreversible intestinal failure. Reported 1‐year, 5‐year, and 10‐year survival rates are 91%, 73%, and 59%, respectively [7, 8], outcomes that are comparable to those of other types of intestinal grafts [9].

Despite significant advances in surgical techniques and perioperative care, postoperative infections remain a leading cause of morbidity and mortality following intestinal transplantation [10, 11]. These complications are strongly linked to ischemia–reperfusion injury (IRI), which disrupts the integrity of the intestinal mucosal barrier, increases epithelial permeability, and facilitates bacterial translocation, thereby exacerbating systemic inflammation and compromising graft function [11, 12]. Among the factors influencing these processes, the type of venous drainage plays a pivotal role in graft homeostasis [12, 13]. Evidence from clinical studies indicates that the maintenance of physiological portal drainage not only supports the immunometabolic integration between the graft and the liver but is also associated with reduced bacterial translocation and lower rates of postoperative infection compared with systemic venous outflow [12, 13]. This protective effect highlights the importance of preserving portal circulation to optimize early graft adaptation, maintain mucosal integrity, and enhance systemic immune regulation.

This study was designed to investigate the acute postoperative phase of living‐donor intestinal transplantation through a controlled 4‐day observation period. This approach enabled a focused assessment of ischemia–reperfusion injury, early immunometabolic responses, and apoptotic modulation, key determinants of short‐term graft viability. While medium‐ and long‐term processes, such as immune adaptation and chronic metabolic changes, were beyond the scope of this model, the study provides a robust platform to elucidate early biological mechanisms that may inform refinements in surgical techniques and perioperative management.

## 2 Materials and Methods

All animals were handled in accordance with the standards for the didactic‐scientific practice of Brazilian vivisection, Law No. 11794, of October 8, 2008, Decree No. 6899, of July 15, 2009, which regulate procedures with animals submitted to scientific research and resolution No. 714 of June 20, 2002 of the Federal Council of Veterinary Medicine (CFMV) which regulates euthanasia procedures. The Ethics Committee for the Use of Animals (CEUA—Faculty of Medicine of USP—SP) approved this project with Protocol number 1355/2019.

Fourteen swine underwent LDIT and were randomized in two groups, receiving portal (portal group: *n* = 7) or systemic (caval group: *n* = 7) venous drainage.

### 2.1 Anesthetic Procedure

Donors and recipients received intramuscular xylazine and ketamine as preanesthetic medication, followed by induction with propofol and orotracheal intubation. Anesthesia was maintained with isoflurane and fentanyl. Intraoperative monitoring included electrocardioscopy, pulse oximetry, capnography, temperature, and blood pressure measurements. A central venous catheter was inserted into the jugular vein for fluid administration and blood gas analysis, while the femoral artery was cannulated for mean arterial pressure monitoring and additional laboratory tests.

### 2.2 Experimental Surgery

The experimental model employed juvenile swine, with a mean body weight of 18–22 kg and age ranging from 8 to 10 weeks. This developmental stage was selected because it is physiologically comparable to human pediatric patients in terms of intestinal length relative to body size, absorptive capacity, hepatic metabolic function, and immune system maturation. These features support the translational relevance of this model for investigating pathophysiological mechanisms and surgical strategies in pediatric intestinal transplantation.

Recipient animals were anesthetized and monitored using the same protocol. For antibiotic prophylaxis, ampicillin (50 mg/kg), cefotaxime (50 mg/kg), and fluconazole (10 mg/kg) were administered intravenously. In both groups, the native intestine was resected while preserving the duodenum and rectum, using a standardized surgical approach.

The surgical procedure for harvesting the donor's small intestine was similar in both groups. Following complete evisceration, the total length of the small intestine was measured. Beginning 20 cm proximal to the ileocecal junction, a 500 cm segment of the small intestine was selected in a retrograde manner. The graft's main artery and vein were identified, and the intestinal segment was subsequently resected.

In the caval group, the donor's inferior vena cava was dissected, and the segment between the renal veins and the iliac vein was removed for use as a venous graft during the recipient's surgery. In portal group, no venous graft was removed from the donor animal.

The intestinal graft was initially perfused with 1 L of cold saline solution supplemented with 5 mL of heparin (5000 IU/mL). This was followed by the infusion of 1 L of HTK (Custodiol) solution via catheterization of the graft artery to flush out the remaining blood supply. After perfusion, the vascular system was rinsed with 250 mL of physiological saline solution containing 50 mL of 20% human albumin. The intestinal lumen was not irrigated.

In both groups, arterial anastomosis was performed using the superior mesenteric artery with a continuous 8–0 Prolene suture. However, the venous anastomosis varied between groups. In the caval group, venous anastomosis was performed end‐to‐side using a venous graft, directly into the recipient's vena cava, with a continuous 7–0 Prolene suture. In the portal group, the venous anastomosis was performed directly to the mesenteric vein using a continuous 7–0 Prolene suture. Prior to the release of the vascular clamps, methylprednisolone (20 mg/kg) was administered to attenuate IRI damage and provide immediate immunosuppression, improving intestinal graft viability.

The duodenum and rectum were anastomosed to the proximal and distal segments of the graft, respectively. The abdominal wall was closed in two layers.

Following surgery, the animals were extubated and transferred to the vivarium for postoperative care. The animals received oral prednisone (1 mg/kg/day) and tacrolimus (1 mg every 12 h). Intravenous administration included saline (0.9%, 100 mL/kg/day), ampicillin (50 mg/kg every 12 h), cefotaxime (50 mg/kg every 12 h), and fluconazole (10 mg/kg/day). Each experiment lasted for 4 days. On the final day, the animals were anesthetized, and liver and intestine biopsies were performed. Subsequently, the animals were euthanized.

Blood samples were collected intraoperatively, as well as on postoperative days one and four. Liver and bowel biopsies were performed following reperfusion and on the fourth postoperative day.

### 2.3 Biochemical and Histological Analyses

Blood samples were collected at anesthetic induction (T0), after reperfusion (T1), 2 h posttransplant (T2), and on the first (T3) and fourth (T4) postoperative days. Biochemical analysis included measurements of albumin, creatinine, urea, ammonia, aspartate aminotransferase (AST), alanine aminotransferase (ALT), gamma glutamyl transferase (GGT), and total proteins.

Histological sections (4 μm) were stained with hematoxylin–eosin (HE) and analyzed under a light microscope. Liver samples were evaluated for inflammation, necrosis, steatosis, and edema, while intestinal samples were assessed for edema, congestion, neutrophil infiltration, goblet cell depletion, epithelial desquamation, loss of villous and crypt architecture, hemorrhage, infarction, and apoptosis. Findings were scored on a scale from 0 (none) to 3 (intense). The slides were analyzed by an experienced pathologist who was blinded to the histological samples.

### 2.4 Immunohistochemistry

Intestinal biopsy fragments were fixed in 10% buffered formaldehyde, embedded in paraffin, and sectioned (4 μm) for caspase‐3 immunohistochemical staining to assess apoptosis. Slides were analyzed at 40× magnification, and caspase‐positive cells were counted in 20 fields. The total count per slide was averaged across the fields.

### 2.5 Molecular Biology Analysis

RNA was extracted from liver and intestine samples, pulverized, and homogenized in TRIZOL. RNA concentration was measured via spectrophotometry, and integrity was assessed by agarose gel electrophoresis. One microgram of RNA was reverse transcribed into cDNA using reverse transcriptase. Gene expression was analyzed by quantitative reverse transcription PCR (qRT‐PCR) for apoptosis‐related genes (Bax, Bcl‐XL), cell proliferation (IL‐6), endothelial function (eNOS), and immune markers (CD8, CXCL10, IFN‐γ, IL‐17, IL‐1a, IL‐1b, TNF‐α, iNOS). β‐actin was used as an internal control. Results were expressed as relative quantification, normalized to health controls.

### 2.6 Statistical Analysis

Continuous variables were summarized as mean ± standard deviation (SD) or median (interquartile range [IQR]), depending on the distribution, which was assessed using the Shapiro–Wilk test. Between‐group comparisons for normally distributed data were performed using independent samples *t*‐tests, while for nonnormally distributed data, the Mann–Whitney *U* test was applied. Categorical variables were compared using chi‐square tests. For time‐series metabolic data, repeated measures analysis of variance (RM‐ANOVA) was conducted, with surgical technique and timepoint as factors. To account for the repeated‐measures design and the interanimal variability inherent to experimental surgical models with limited sample sizes, linear mixed models (LMM) were applied. The LMM analysis was performed specifically to validate and refine the interpretation of results that showed statistical significance in the initial repeated‐measures ANOVA.

Principal Component Analysis (PCA) was used to identify biological differences between surgical techniques, incorporating immunological, metabolic, molecular, and pathological markers, highlighting the directions of greatest variance, allowing the elimination of components without a significant impact on the composition of the data. The top three principal components were retained based on variance explained. Group differences in PC1 and PC2 scores were assessed using Mann–Whitney *U* tests, and Cohen's *d* effect sizes were calculated.

All statistical analyses were performed using Python (version 3.9.6), with the following libraries: pandas (data preprocessing and management), SciPy (statistical testing), Scikit‐learn (PCA computation and feature scaling), Statsmodels (RM‐ANOVA), and Matplotlib and Seaborn (data visualization).

A two‐tailed *p*‐value < 0.05 was considered statistically significant for all comparisons.

## 3 Results

### 3.1 Demographic Characteristics of the Study Population

The experiment lasted 4 days, during which all transplanted animals remained in a healthy condition. Notably, no cases of vascular thrombosis were observed in any of the 14 transplants performed. No significant differences were observed in donor weight (*p* = 0.2613), recipient weight (*p* = 0.6002), cold ischemia time (*p* = 0.2814), warm ischemia time (*p* = 0.7569), or graft length (*p* = 0.0941) between groups (Table 1).

**TABLE 1:** Demographic characteristics of the study population.

Variable	Portal group (Mean ± SD)	Caval group (Mean ± SD)	*p*
Donor weight (kg)	42.43 ± 3.51	40.00 ± 4.16	0.2613
Recepient weight (kg)	22.26 ± 2.77	21.57 ± 1.90	0.6002
Cold Ischemia (min)	141.86 ± 19.89	161.00 ± 39.43	0.2814
Warm ischemia Warm (min)	32.00 ± 6.53	33.14 ± 6.96	0.7569
Graft Length (cm)	464.29 ± 47.56	500.00 ± 0.00	0.0941
Graft Ratio, cm/kg	21.02 ± 2.54	23.33 ± 2.04	0.0860

Abbreviation: SD, standard deviation.

### 3.2 Metabolic Marker Analysis

**FIGURE 1:**
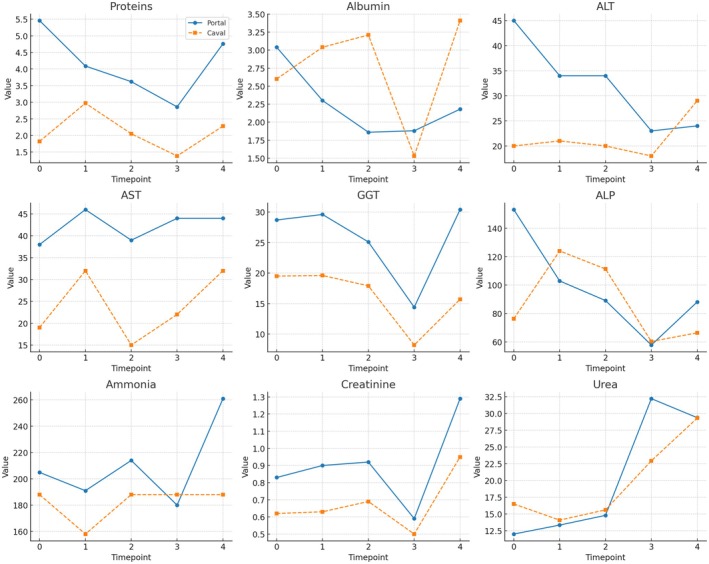
Longitudinal biochemical kinetics in portal vs. caval drainage groups after living donor intestinal transplantation. Time‐course of serum biochemical parameters in two experimental groups: portal venous drainage (blue solid line; *n* = 7) and systemic (caval) drainage (orange dashed line; *n* = 7). Panels show Proteins, Albumin, ALT (alanine aminotransferase), AST (aspartate aminotransferase), GGT (gama‐glutamyl transferase), ALP(alkaline phosphatase), Ammonia, Creatinine, and Urea. Measurements were obtained at five standardized timepoints: T0 – preoperative; T1 – immediately after reperfusion; T2 – 2 h post‐reperfusion; T3 – postoperative day 1; and T4 – postoperative day 4. Data are expressed as mean ± SD.

**FIGURE 2:**
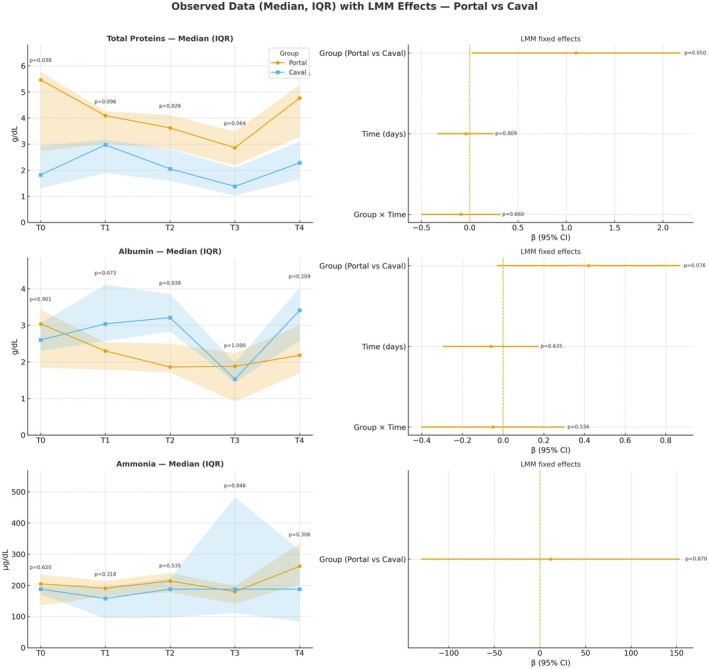
Temporal kinetics and mixed‐model analysis of serum proteins, albumin, and ammonia in portal and systemic drainage groups after living donor intestinal transplantation. Temporal evolution of median and interquartile range (IQR) of total proteins, albumin, and ammonia in swine recipients with portal venous drainage (blue) and systemic (caval) drainage (orange) across five standardized timepoints: T0 – preoperative; T1 – immediately after reperfusion; T2 – 2 h post‐reperfusion; T3 – postoperative day 1; and T4 – postoperative day 4. Shaded areas represent the IQR. Right panels present fixed effects from linear mixed‐effects models (LMM), expressed as β coefficients with 95% confidence intervals (CI), assessing the influence of Group (portal vs. caval), Time (days), and their interaction (Group × Time). Relevant *p*‐values are indicated for intergroup and temporal comparisons.

Biochemical analysis revealed no significant differences between groups in classical liver function markers, including ALT, AST, ALP, GGT, ammonia, creatinine, and urea, across all timepoints (*p* > 0.05). However, significant differences were observed in serum protein profiles. Total serum protein levels were consistently higher in the portal drainage group at baseline (T0: 5.46 vs. 1.82 g/dL, *p* = 0.037), on postoperative day 2 (T2: 3.62 vs. 2.05 g/dL, *p* = 0.026), and on postoperative day 4 (T4: 4.76 vs. 2.28 g/dL, *p* = 0.0262) compared to the systemic group. Additionally, serum albumin was significantly higher in the systemic drainage group on postoperative day 2 (T2: 3.21 vs. 1.86 g/dL, *p* = 0.037), although no consistent trend was observed across other timepoints. Linear mixed model (LMM) analysis revealed that total protein levels were consistently higher in the Portal groupc ompared with the Caval group across all experimental time points (*β* = +1.10 g/dL; 95% CI: 0.00 to 2.20; *p* = 0.050), indicating a borderline statistically significant effect. Albumin levels showed no significant differences between groups (*β* = +0.42 g/dL; 95% CI: −0.04 to 0.88; *p* = 0.076), although slightly higher values were noted in the Caval group at certain time points. For globulin levels, calculated as the difference between total protein and albumin, there was a nonsignificant trend toward higher concentrations in the Portal group (*β* = +0.54 g/dL; 95% CI: −0.26 to 1.34; *p* = 0.193). These results indicate a consistent pattern of higher protein synthesis markers in the Portal group, albeit with variable statistical significance across parameters. These results indicate a consistent pattern of elevated protein synthesis markers in the Portal group during the early postoperative phase. For Ammonia, Linear mixed‐effects modeling revealed no significant group effect (*β* = +11.9 μmol/L; 95% CI: −130.1 to +153.9; *p* = 0.870) and no significant group × time interaction across the study period. Similarly, no consistent temporal effect was observed at T1 (*β* = −45.7 μmol/L; 95% CI: −447.3 to +355.9; *p* = 0.823), T2 (*β* = −17.0 μmol/L; 95% CI: −418.6 to +384.6; *p* = 0.934), T3 (*β* = +105.6 μmol/L; 95% CI: −296.1 to +507.2; *p* = 0.606), or T4 (*β* = +47.8 μmol/L; 95% CI: −353.8 to +449.4; *p* = 0.816). While descriptive statistics showed a slightly higher mean in the Portal group at T4 (285.3 μmol/L vs. 242.8 μmol/L in the Caval group), the broad confidence intervals and nonsignificant interaction term (*β* = +30.6 μmol/L; 95% CI: −170.2 to +231.5; *p* = 0.765) indicate that this observation likely represents an isolated finding rather than a consistent longitudinal difference in ammonia dynamics between venous drainage techniques (Figures 1 and 2).

### 3.3 Pathology

Liver histology did not demonstrate statistically significant differences between portal and caval groups across all evaluated parameters (*p* > 0.05). Mild portal inflammation was observed more frequently in the portal group (0.71 ± 0.76) compared to the caval group (0.33 ± 0.58; *p* = 0.529). Lobular inflammation was present only in the portal group (0.43 ± 0.53 vs. 0.00 ± 0.00; *p* = 0.253), whereas hepatocellular ballooning was observed only in the caval group (0.33 ± 0.58 vs. 0.00 ± 0.00; *p* = 0.190). No lobular necrosis or portal fibrosis was detected in either group (both *p* = 1.000). Hepatic congestion was mild and similar between groups (portal: 0.86 ± 0.90; systemic: 0.67 ± 0.58; *p* = 0.903). These results indicate preserved hepatic architecture and absence of significant injury or fibrotic response during the early postoperative period.

Histological analysis of intestinal tissue showed no statistically significant differences between the groups across the evaluated parameters (*p* > 0.05). Epithelial denudation was present only in the portal group (1.14 ± 1.35 vs. 0.0 ± 0.0; *p* = 0.65), and villous blunting was also more pronounced in the portal group (1.43 ± 1.40 vs. 0.8 ± 1.3; *p* = 0.54). In contrast, lymphocytic epithelial infiltration was more frequent in the caval group (1.0 ± 0.71) compared to the portal group (0.29 ± 0.49), with a trend toward significance (*p* = 0.086), suggesting a potentially stronger local immune response in the caval group. Mucosal apoptosis, crypt reactivity, and vascular inflammation were absent in both groups (0.0 ± 0.0; *p* = 1.0), and vascular congestion was comparable (portal: 0.57 ± 0.79; systemic: 0.6 ± 0.55; *p* = 0.857). These findings indicate minimal structural injury and support an overall comparable pattern of intestinal mucosal preservation between groups in the early postoperative period (Tables 2 and 3).

**TABLE 2:** Liver pathological feature.

Pathological feature	Portal (Mean ± SD)	Caval (Mean ± SD)	*p*
Portal inflammation	0.71 ± 0.76	0.33 ± 0.58	0.529
Lobular necrosis	0.00 ± 0.00	0.00 ± 0.00	1.000
Portal fibrosis	0.00 ± 0.00	0.00 ± 0.00	1.000
Lobular inflammation	0.43 ± 0.53	0.00 ± 0.00	0.253
Hepatocellular ballooning	0.00 ± 0.00	0.33 ± 0.58	0.190
Congestion	0.86 ± 0.90	0.67 ± 0.58	0.903

**TABLE 3:** Intestinal pathological feature.

Intestinal pathological feature	Caval group (Mean ± SD)	Portal group (Mean ± SD)	*p*
Epithelial denudation	0.0 ± 0.0	1.14 ± 1.35	0.65
Mucosal apoptosis	0.0 ± 0.0	0.0 ± 0.0	1.00
Flattening of Villi	0.8 ± 1.3	1.43 ± 1.4	0.54
Crypt reactivity	0.0 ± 0.0	0.0 ± 0.0	1.00
Lymphocyte epithelial permeation	1.0 ± 0.71	0.29 ± 0.49	0.08
Inflamação em inflammation in vessels	0.0 ± 0.0	0.0 ± 0.0	1.00
Vascular congestion	0.6 ± 0.55	0.57 ± 0.79	0.85

### 3.4 Immunohistochemistry

**FIGURE 3:**
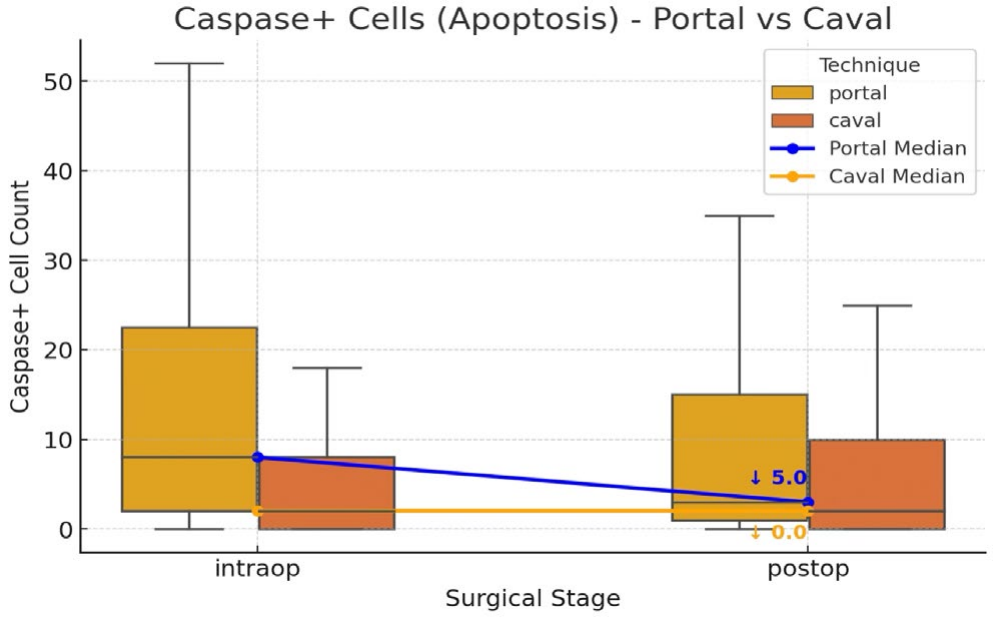
Intracellular caspase analysis. Box plots illustrate the distribution of Caspase+ cell counts (apoptosis marker) measured intraoperatively (intraop) and postoperatively (postop), comparing the portal (blue) and caval (orange) surgical techniques. The central line indicates the median, and the box denotes the interquartile range (IQR, Q1–Q3). Overlaid lines represent paired median trajectories, highlighting intrapatient changes from intraoperatively to postoperatively.

Intraoperative caspase‐positive cell counts were significantly higher in the portal group compared to the caval group (median 8.00 [IQR: 2.00–22.50] vs. 2.00 [IQR: 0.00–8.00]; *p* < 0.0001). Although postoperative caspase counts remained elevated in the portal group (3.00 [IQR: 1.00–15.00] vs. 2.00 [IQR: 0.00–10.00]), this difference did not reach statistical significance (*p* = 0.0553). Within‐group analysis showed a significant reduction in caspase expression from intraoperative to postoperative timepoints in the portal group (*p* = 0.0044), while no such difference was observed in the caval group (*p* = 0.8885) (Figure 3).

### 3.5 Molecular Marker Analyses

Proapoptotic (BAX) and anti‐apoptotic (BclXL) markers showed stable expression levels between groups, both intraoperatively (BAX: 0.08 ± 0.07 vs. 0.07 ± 0.03; *p* = 0.5975) and postoperatively (BclXL: 0.46 ± 0.98 vs. 0.10 ± 0.09; *p* = 0.4037), indicating no significant shift in apoptotic regulation at the hepatic level. Markers of immune activation, including CD8, CXCL10, IL1a, IL6, and TNFα, exhibited variable expression across groups and timepoints, but without significant group differences. Notably, CD8+ T cell infiltration tended to be higher in the systemic group postoperatively (7.34 ± 7.61 vs. 4.04 ± 1.72), although this was not statistically significant (*p* = 0.3371). Inflammatory cytokines such as IL17, IFNγ, and iNOS demonstrated elevated trends in the systemic group postoperatively (e.g., IL17: 8.51 ± 7.84 vs. 3.65 ± 3.03), again without reaching significance (*p* = 0.1964). These findings suggest comparable inflammatory and apoptotic profiles in hepatic tissue between the portal and caval drainage groups during the early postoperative phase.

Molecular analysis of intestinal tissue revealed largely comparable expression profiles between the portal and caval drainage groups, with most markers showing no statistically significant differences (*p* > 0.05). Proapoptotic BAX and anti‐apoptotic BclXL expression levels were similar at both timepoints, with no significant intra‐ or inter‐group variation (e.g., postoperative BAX: 0.75 ± 0.68 vs. 0.57 ± 0.30; *p* = 0.5534).

Markers of cytotoxic lymphocyte activity, such as CD8, were higher in the caval group at both timepoints, particularly postoperatively (1.46 ± 1.41 vs. 0.45 ± 0.33; *p* = 0.1347), though this did not reach significance. Similarly, CXCL10, a chemokine linked to T‐cell recruitment, also showed a nonsignificant increase in the caval group (postoperative: 0.64 ± 0.81 vs. 0.38 ± 0.25; *p* = 0.4687).

**FIGURE 4:**
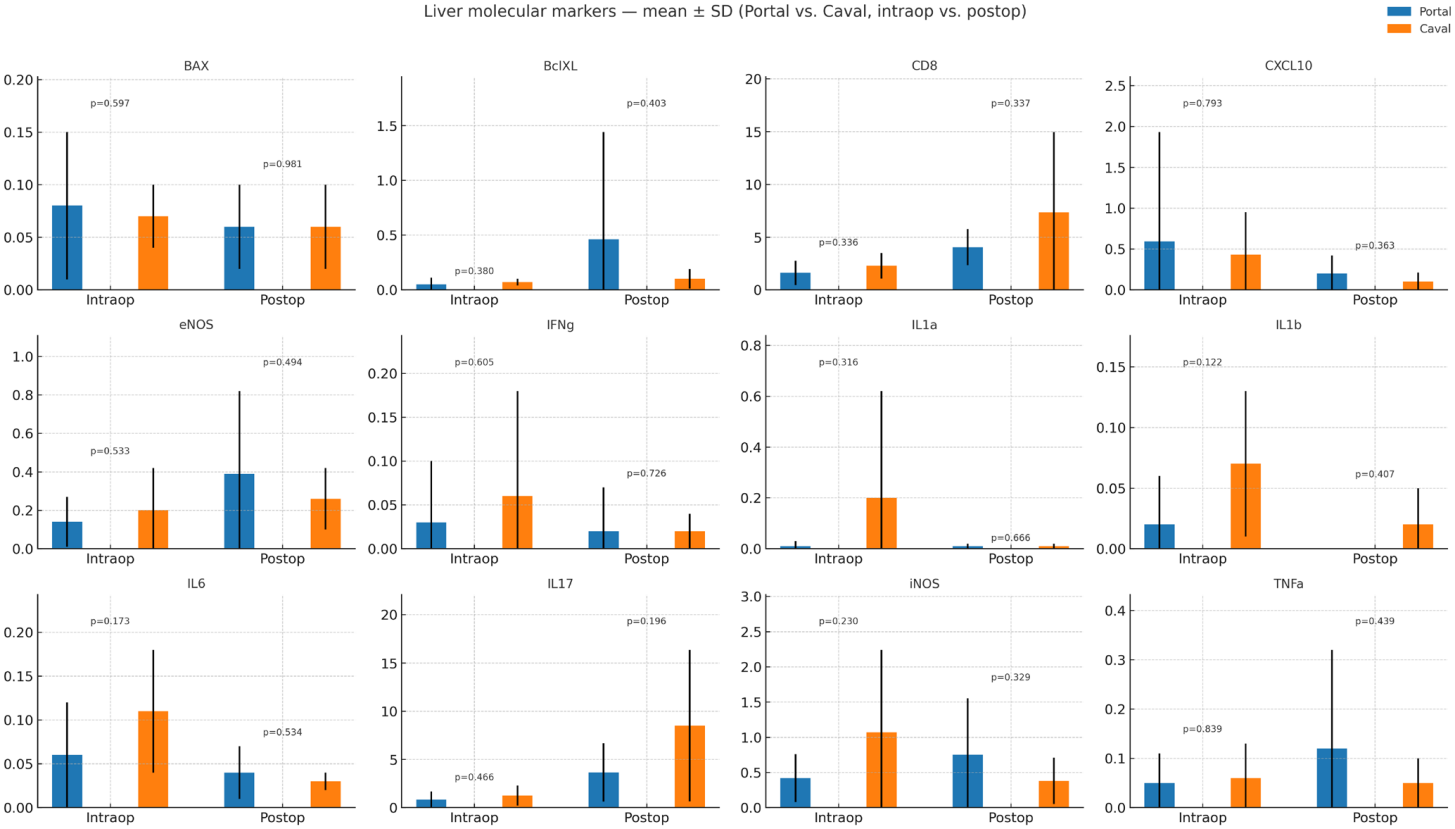
Hepatic molecular expression profiles in portal and systemic drainage groups after living donor intestinal transplantation. Bar plots show the mean ± SD expression of apoptotic (BAX, Bcl‐XL), inflammatory (IL‐1α, IL‐1β, IL‐6, IL‐17, TNF‐α, IFN‐γ, CD8), and endothelial/oxidative (eNOS, iNOS, CXCL10) markers in liver tissue of swine recipients undergoing portal venous drainage (blue) or systemic (caval) drainage (orange). Measurements were obtained at two standardized timepoints: Intraop – immediately after reperfusion; and Postop – postoperative day 4. Bars represent group means with standard deviation, and *p*‐values are indicated for intergroup comparisons at each timepoint. Portal drainage was associated with a trend toward higher anti‐apoptotic (Bcl‐XL) and lower pro‐inflammatory (IL‐1β, IL‐17, TNF‐α) expression, suggesting more balanced regulation of inflammatory and apoptotic pathways compared with systemic drainage.

**FIGURE 5:**
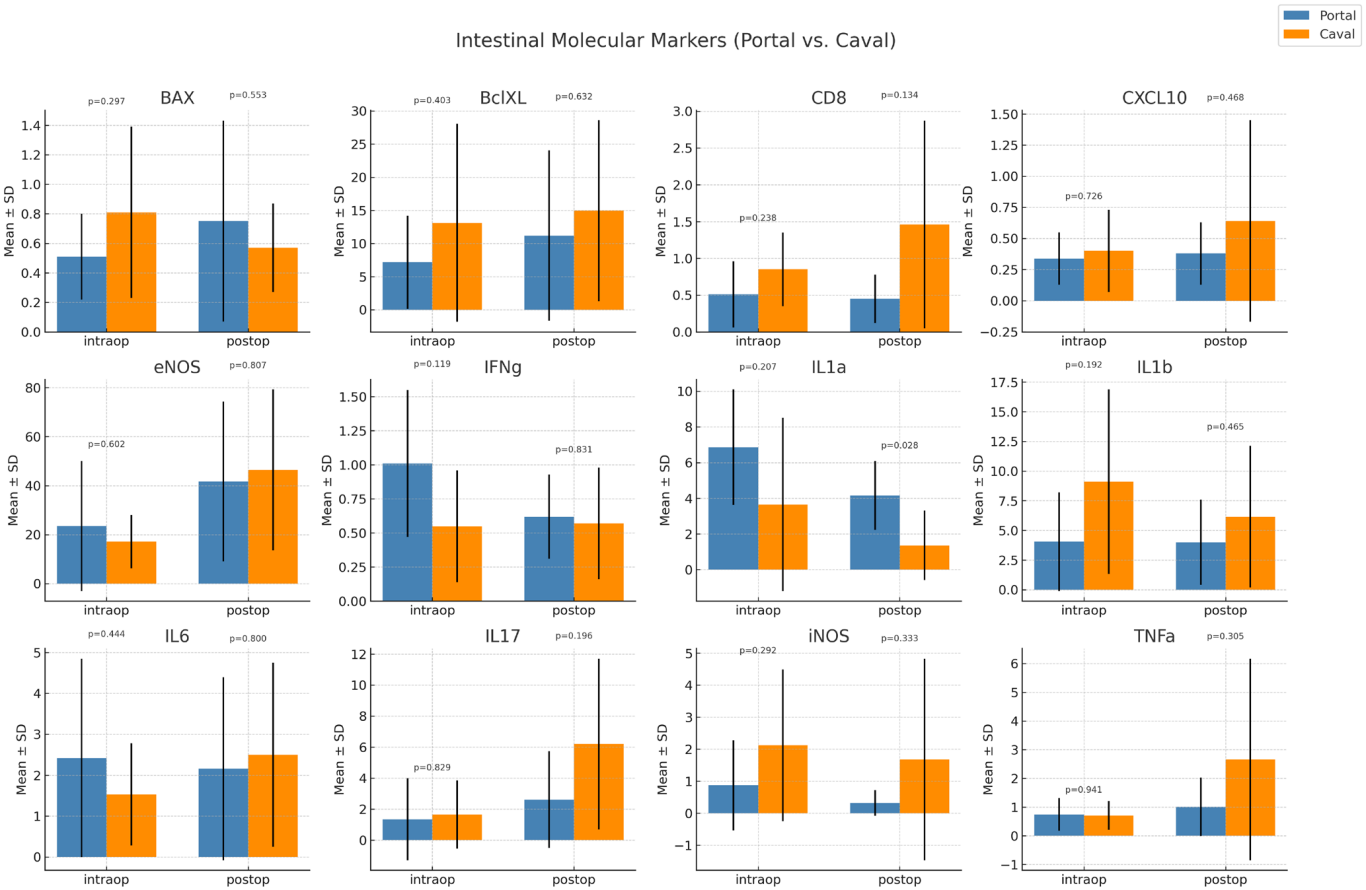
Intestinal molecular expression profiles in portal and systemic drainage groups after living donor intestinal transplantation. Bar plots display the mean ± SD expression of apoptotic (BAX, Bcl‐XL), inflammatory (IL‐1α, IL‐1β, IL‐6, IL‐17, TNF‐α, IFN‐γ, CD8), and endothelial/oxidative (eNOS, iNOS, CXCL10) markers in intestinal tissue of swine recipients submitted to portal venous drainage (blue) or systemic (caval) drainage (orange). Samples were collected at two standardized timepoints: Intraop – immediately after reperfusion; and Postop – postoperative day 4. Bars represent group means with standard deviation, and *p*‐values indicate intergroup comparisons at each timepoint.

A notable finding was the significantly higher expression of IL1α in the portal group postoperatively (4.16 ± 1.93 vs. 1.36 ± 1.95; *p* = 0.028), suggesting enhanced innate immune activation or epithelial signaling in this group. Other inflammatory cytokines—including IFNγ, IL1β, IL6, IL17, and TNFα—exhibited heterogeneous but nonsignificant differences between groups at both timepoints. Finally, iNOS and eNOS, markers of nitric oxide signaling and vascular response, did not differ significantly, although iNOS tended to be elevated in the caval group postoperatively (1.68 ± 3.15 vs. 0.32 ± 0.40; *p* = 0.3332) (Figures 4 and 5).

### 3.6 Principal Component (PC) Analysis

- PC1, accounting for 21.4% of the variance, was primarily associated with immune‐inflammatory markers, particularly IL17 and CD8 in both liver and intestinal tissues, as well as total protein levels, suggesting that cytotoxicity and inflammation as key differentiating factors.
- PC2, explaining 15.35% of the variance, was driven by inflammatory and apoptotic markers, including IFNg, CXCL10, BAX, GGT, and iNOS, indicating tissue damage and immune activation.
- PC3, which accounted for 12.47% of the variance, was linked to epithelial injury and hepatic stress, with major contributors including epithelial denudation, BclXL, ALT, eNOS, and lymphocyte infiltration.

**FIGURE 6:**
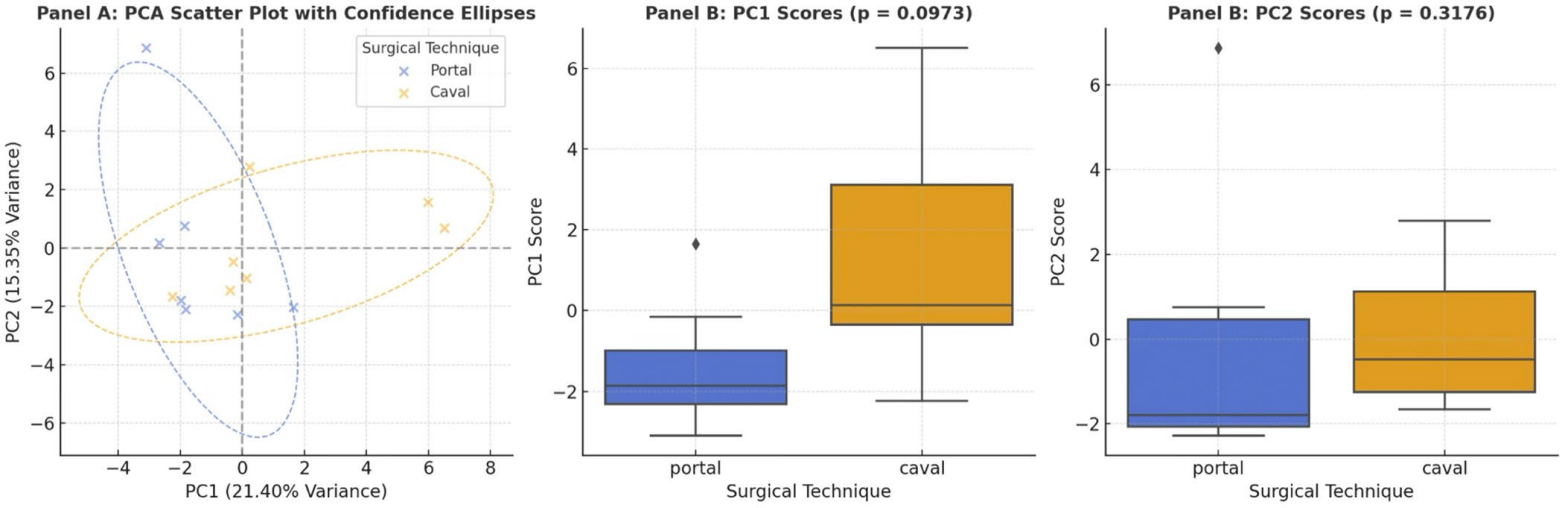
Principal component analysis (PCA) of biochemical, molecular, and histological biomarkers comparing portal and systemic drainage groups. Panel A: Two‐dimensional representation of the first two principal components (PC1 and PC2), which together explain 36.75% of the total variance (PC1 = 21.4%; PC2 = 15.35%). Each point represents one animal, color‐coded by the surgical technique (blue = portal drainage; orange = caval drainage). The spatial proximity of points reflects greater similarity in biological profiles. Panel B: Distribution of PC1 scores according to drainage technique. Higher PC1 values indicate stronger contributions of biomarkers with positive PC1 loadings. Panel C: Distribution of PC2 scores by drainage technique, illustrating variability explained by the secondary component. The analysis highlights clustering trends between groups, supporting the influence of venous drainage route on global immunometabolic signatures.

**FIGURE 7:**
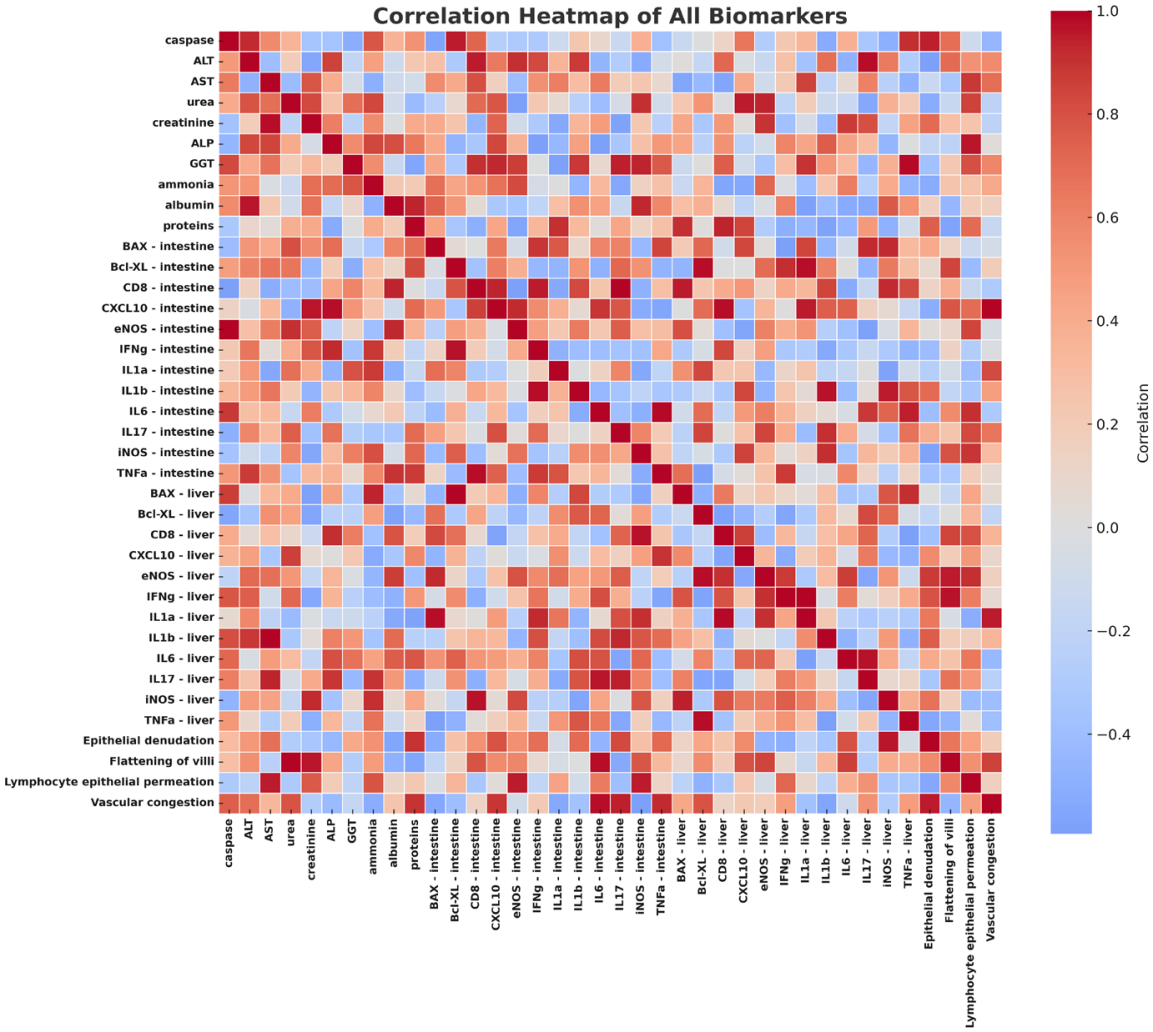
Correlation map of biochemical, molecular, and histological biomarkers in the experimental model. Heatmap illustrating the Pearson correlation matrix among all evaluated biomarkers. Each row and column corresponds to an individual biomarker, while the color scale indicates the strength and direction of correlation coefficients: dark red (+1.0) denotes strong positive correlation (both variables increase together); dark blue (–1.0) denotes strong negative correlation (inverse relationship); and light tones (~0.0) represent weak or no significant correlation. The diagonal represents the perfect self‐correlation (+1.0) of each variable. This visualization enables identification of biological clusters and interaction patterns among inflammatory, metabolic, and molecular pathways observed in the experimental model, highlighting coordinated responses related to ischemia–reperfusion and venous drainage profiles.

PCA revealed distinct biological patterns between the surgical techniques, primarily driven by immune‐inflammatory and metabolic markers (IL17, CD8, IFNg, CXCL10, proteins, GGT, ALT). The portal group exhibited lower scores on PC1 (*p* = 0.0973), suggesting a tendency toward reduced inflammatory and metabolic stress compared to the cava group. No significant differences were found on PC2 (apoptotic axis, *p* = 0.3176), indicating similar levels of apoptosis and liver injury between groups (Figures 6 and 7).

## 4 Discussion

The disruption of the intestinal mucosal barrier secondary to ischemia–reperfusion injury (IRI) is a pivotal event in the pathogenesis of early complications after intestinal transplantation. Experimental models consistently demonstrate that IRI initiates a complex cascade involving microcirculatory dysfunction, endothelial activation, leukocyte recruitment, and oxidative stress, leading to loss of epithelial integrity, increased mucosal permeability, and facilitation of bacterial translocation [14, 15]. The preservation of portal venous outflow during transplantation is widely recognized as essential for maintaining physiological graft perfusion, and emerging evidence suggests it may exert a modulatory effect on immune regulation and tissue repair, thereby potentially reducing the risk of infection‐related complications [12, 13]. Although our model was restricted to a four‐day observation period, this design was intentionally focused on the acute postoperative phase, which is characterized by intense ischemia–reperfusion injury and the activation of early immune and metabolic pathways that are critical for graft viability. While medium‐ and long‐term processes, such as immune adaptation and chronic mucosal remodeling, were beyond the scope of this study, our findings provide important mechanistic insights into the early events that shape graft adaptation and may inform strategies to optimize perioperative management and improve clinical outcomes in intestinal transplantation.

In this context, our findings support and extend prior observations by Hashimoto et al. [16] and Barney et al. [12, 13], who demonstrated comparable patient and graft survival regardless of the venous drainage technique. Similarly, in our model, conventional biochemical markers of hepatic function remained preserved in both groups during the early postoperative period, reinforcing that the type of venous outflow does not critically affect short‐term liver function. This suggests that potential differences between portal and systemic drainage may emerge not at the level of global liver function, but rather through more subtle mechanisms of immune, apoptotic, and metabolic modulation, as explored in the present study.

The trend toward higher total protein and globulin levels in the Portal group, although influenced by baseline variability, is biologically plausible and aligns with the physiological role of portal venous flow in delivering hepatotrophic factors and substrates to the liver. By sustaining this physiological integration, portal drainage may promote a more favorable metabolic environment in the immediate postoperative period, supporting protein synthesis and systemic immune balance. While the modest magnitude of the effect and the variability observed underscore the need for cautious interpretation, these findings suggest that subtle metabolic advantages of portal drainage may contribute to early graft adaptation and could translate into clinically meaningful outcomes if confirmed in larger experimental cohorts.

In the context of intestinal transplantation, elevated globulin levels may reflect an enhanced hepatic synthetic response or an upregulation of protective immunoglobulin production, potentially contributing to improved inflammatory regulation and bacterial clearance [17, 18]. Thus, the maintenance of physiological portal flow may offer not only perfusion benefits but also metabolic and immunological advantages that warrant further investigation for their potential impact on clinical outcomes.

Histological evaluation demonstrated only mild, early‐grade alterations consistent with Chiu/Park [19] grades 1–2, indicating that ischemia–reperfusion injury was largely confined to superficial mucosal layers without progression to advanced destructive patterns. Importantly, when interpreted according to the criteria of Lee et al. [20] for acute rejection, no features of severe rejection were observed, as crypt architecture and epithelial integrity were preserved in all cases. A near‐significant trend toward greater lymphocytic infiltration in the Caval group suggests enhanced local immune activation in the absence of physiological portal drainage, yet this pattern is more consistent with early ischemia–reperfusion–related immune stress rather than rejection. Although we did not perform direct assessments of bacterial translocation, endotoxemia, intestinal permeability, or graft hemodynamics, the overall data provide indirect evidence that portal drainage may support mucosal barrier function and early immunometabolic balance, as reflected by modulation of inflammatory and apoptotic pathways, a trend toward higher globulin synthesis, and reduced lymphocytic infiltration. Taken together, these findings indicate that the venous drainage route does not determine overt structural injury in the immediate postoperative period but may influence early immune regulation at a subclinical level, a hypothesis that warrants confirmation in future studies incorporating direct microbiological, permeability, and microcirculatory analyses.

Immunohistochemistry for caspase provided a sensitive readout of early apoptotic activation, capable of detecting molecular events not yet apparent on conventional histology [21, 22, 23]. This methodological sensitivity likely explains why apoptotic activity was demonstrable in our analysis despite the absence of morphologically evident apoptosis in routine staining [24]. Our findings raise the hypothesis that physiological portal flow may trigger an early, adaptive apoptotic response during reperfusion, functioning as a controlled mechanism to limit progression to necrosis and thereby contribute to the preservation of mucosal integrity.

As the postoperative course progressed, a distinct temporal pattern of apoptotic modulation emerged. Whereas caspase activity remained relatively stable in the systemic group, the portal group exhibited a marked reduction, raising the hypothesis that physiological portal drainage facilitates a more efficient downregulation of apoptosis once the initial reperfusion insult has subsided. Such a dynamic profile suggests that portal outflow may not only trigger an early apoptotic response during reperfusion but also promote its timely resolution, thereby supporting the restoration of mucosal homeostasis and potentially limiting excessive tissue damage.

Because excessive apoptosis compromises epithelial integrity and facilitates bacterial translocation, the capacity to rapidly reestablish apoptotic balance may be critical for graft protection and infection control. In this regard, the decline in caspase activity observed in the portal group, in parallel with stable histological preservation and enhanced IL‐1α expression, supports the hypothesis of a coordinated immunometabolic response promoted by physiological portal flow. At the molecular level, although classical apoptotic markers such as BAX and Bcl‐XL did not differ significantly between groups, the immunohistochemical evidence underscores the value of caspase staining as a sensitive indicator of early epithelial stress, capturing dynamic changes not readily detected by routine histology or gene expression alone.

One of the most distinctive findings of this study was the selective upregulation of interleukin‐1α (IL‐1α) in the portal group during the immediate postoperative phase. IL‐1α plays a pivotal role in preserving intestinal barrier integrity by stimulating epithelial adhesion molecules and signaling pathways that reinforce intercellular cohesion, thereby reducing paracellular permeability and limiting bacterial translocation [25, 26]. Beyond its epithelial effects, IL‐1α also promotes neutrophil and macrophage activation, facilitating efficient clearance of microbial products and containment of local infections [25, 26, 27]. The hypothesis emerging from our data is that physiological portal drainage favors an IL‐1α‐mediated immunometabolic adaptation, in which the early enhancement of innate immune signaling contributes to mucosal stability and protection against systemic bacterial dissemination. In contrast, this protective cytokine response was not evident in the systemic drainage, suggesting that venous outflow configuration may influence the trajectory of early immune activation.

These findings suggest that the preservation of portal flow may facilitate a local microenvironment that favors immune coordination and epithelial defense. When integrated with our immunohistochemical results—demonstrating a rapid reduction in caspase‐mediated apoptosis in the same group—and the elevated globulin concentrations observed in serum, the increased IL‐1α expression further supports the hypothesis that portal drainage contributes to a proresolving and immunoprotective profile. Thus, IL‐1α may act as both a biomarker and an effector of improved mucosal resilience in this setting, representing a key molecular signature of the physiological benefits associated with portal venous drainage in intestinal transplantation.

To integrate the diverse datasets and capture broader biological patterns, we employed Principal Component Analysis (PCA). This multivariate approach highlighted that portal and systemic drainage groups differed mainly in immune‐inflammatory and metabolic dimensions. The systemic group was associated with stronger activation of IL‐17 and CD8 responses, suggesting an intensified cytotoxic profile, whereas the portal group clustered toward a less activated inflammatory state and higher serum protein synthesis. These trends are consistent with the observed reduction in lymphocytic infiltration, downregulation of caspase activity, and higher globulin production in the portal group, pointing to a more balanced immunometabolic environment. Additional components of the PCA underscored that both drainage strategies preserved hepatic integrity and apoptotic equilibrium within the short observation window, while subtle trends suggested that portal drainage may attenuate epithelial stress and support faster mucosal recovery. Taken together, these multivariate findings reinforce the hypothesis that physiological portal flow favors coordinated immune modulation and tissue protection.

## 5 Conclusion

This study shows that venous drainage configuration shapes early biological responses in living‐donor intestinal transplantation. Portal drainage was associated with a less inflammatory and more balanced immunometabolic profile, including early but controlled apoptosis and selective IL‐1α upregulation. These findings, while exploratory, highlight the potential protective role of portal flow in graft adaptation and warrant further investigation in extended models.

## Author Contributions

Guilherme F. Paganoti: Contributed to the research design, manuscript writing, research execution, and data collection and analyses. Ana Cristina A. Tannuri: Contributed to the research design, manuscript writing, and research execution. Uenis Tannuri: contributed to the research design and manuscript writing. Alessandro R. Belon: Contributed to the research design. Josiane O. Gonçalves: Contributed to the research execution. Suellen Serafin: Contributed to the research. Raimundo R.N. Guimarães: Contributed to the research. Felipe Y. Matsushita: Contributed to data analyses.

## Conflicts of Interest

The authors declare no conflicts of interest.

## Data Availability

The data that support the findings of this study are available from the corresponding author upon reasonable request.
